# Open questions: missing pieces from the immunological jigsaw puzzle

**DOI:** 10.1186/1741-7007-11-10

**Published:** 2013-01-31

**Authors:** Gillian M Griffiths

**Affiliations:** 1 Cambridge Institute for Medical Research, Hills Rd, Cambridge, CB2 0XY, UK

## 

There is nothing more frustrating than reaching the end of a jigsaw puzzle to find that some of the pieces are missing. There are certainly areas of immunology where the same problem applies and despite major advances there are some pieces of the jigsaw that are still missing after many years. When I was asked to think of some of the questions that remain unanswered in the cell biology of the immune system, three leapt to mind. One concerns antigen presentation, another cytotoxic T lymphocyte (CTL)- or natural killer (NK)-cell-mediated killing, and the third a mechanism of apoptosis. Although seemingly disparate, each one of these boils down to a question of how proteins cross membranes to reach the cytoplasm.

## The missing step that allows 'cross-priming'

**Figure 1:**
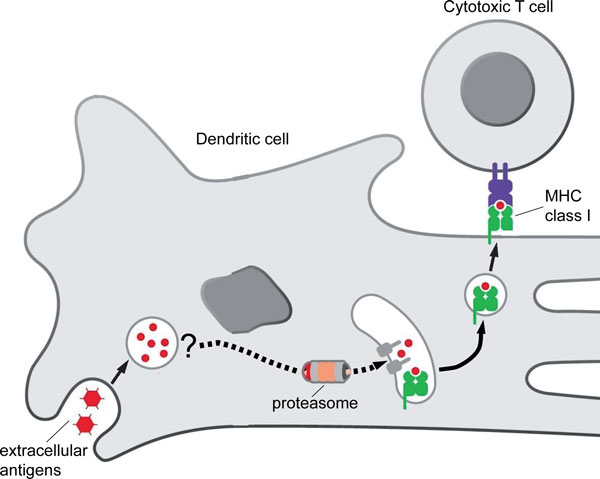
**Cross-presentation of extracellular antigen to a cytotoxic T cell by an activating dendritic cell**. Adapted from Figure 4-20 in DeFranco AL, Locksley RM, Robertson M *Immunity: The Immune Response in Infectious and Inflammatory Disease*. London: New Science Press; 2007.

The first is the mechanism that lies at the heart of 'cross-presentation' [1] and allows extracellular antigens to be endocytosed by dendritic cells, released into the cytoplasm, and displayed to cytotoxic T lymphocytes through the proteasomal/MHC class I pathway that operates in the presentation of intracellular antigens (Figure 1). Although it has been clear for many years that proteins are released from endocytic compartments into the cytoplasm [2], where they are exposed to the proteasomal pathway, the mechanism and trigger for this critical transport step remain unclear.

## How do cytotoxic cells avoid self-destruction?

**Figure 2:**
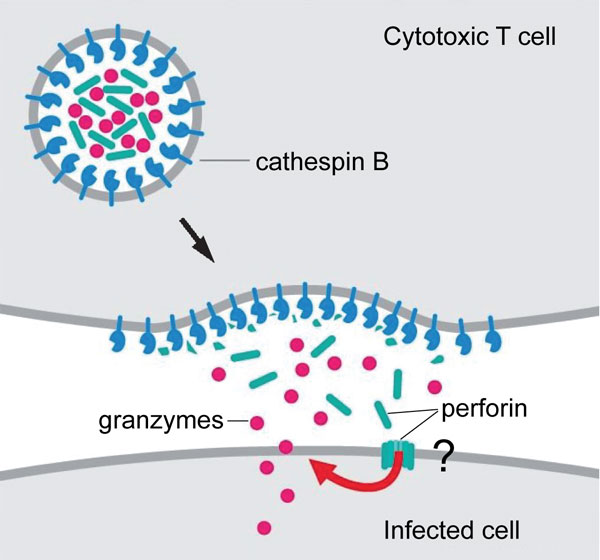
**Perforin release from a cytotoxic cell in the presence of cathepsin B**. Adapted from Figure 5-35 in DeFranco AL, Locksley RM, Robertson M *Immunity: The Immune Response in Infectious and Inflammatory Disease*. London: New Science Press; 2007.

The second problem, which remains much debated over many years, concerns how and where the pore-forming protein perforin, used by CTL and NK cells to destroy their targets, does or does not cross membranes. A great deal of research has focused on whether perforin forms its pore as it reaches the plasma membrane or is first taken up into endosomes. However a knottier and much less studied problem is how CTLs resist killing by perforin as they kill their targets. Why is the integrity of the CTL plasma membrane not compromised during target cell killing? There is no innate mechanism of resistance in the plasma membrane, since CTLs can themselves be targets. To date the only mechanism proposed for this resistance is a protective coating of cathepsin B (capable of cleaving perforin and rendering it non-functional) on the inside of the secretory granules from which perforin is released [3] (Figure 2). However, since cathepsin B-deficient CTL do not self-destruct upon killing [4], it is clear that this cannot be the only protective mechanism in play.

## A chicken-and-egg problem in apoptosis

The third problem concerns apoptosis. One recently proposed pathway of caspase-independent apoptosis [5] suggests that the initial event triggering cell death is rupture of the lysosomal membranes releasing cathepsins that can be active in the cytoplasm. This pathway is thought to provide an additional mechanism for triggering apoptosis. There is something of a chicken-and-egg problem, however, as it is difficult to be sure that lysosomal breakdown is the initiating event rather than a consequence of rapid cell death. However, the key question that remains is how the membrane is ruptured to allow the proteins to escape into the cytoplasm.
